# Pus-Forming Infections in an Advanced Care Hospital in Morocco: Enterobacterales Dominance With High Burden of Extended-Spectrum β-Lactamase Producers and Carbapenem Resistance

**DOI:** 10.7759/cureus.103918

**Published:** 2026-02-19

**Authors:** Youssef Benoumrhar, Yousra Boughalem, Youssef El Kamouni, Said Zouhair, Lamiae Arsalane

**Affiliations:** 1 Department of Microbiology, Avicenna Military Hospital, Marrakech, MAR; 2 Faculty of Medicine and Pharmacy, Cadi Ayyad University, Marrakech, MAR

**Keywords:** antimicrobial resistance, antimicrobial susceptibility, carbapenem resistance, enterobacterales, extended-spectrum beta-lactamase, hospital-acquired infection, pus-forming infections

## Abstract

Background and aim

Pus-forming infections are a common cause of morbidity and are increasingly complicated by rising antimicrobial resistance (AMR). This study aimed to describe the bacteriological profile and antimicrobial resistance patterns of bacteria isolated from specimens in an advanced care hospital in Morocco, with a focus on multidrug resistant gram negative bacilli.

Methods

We conducted a single-centre, observational cross-sectional study from July 2024 to October 2025 in an advanced care hospital in Marrakech, Morocco. All pus specimens (fine-needle aspirates and wound swabs) received in the microbiology laboratory were processed using standard culture methods. Isolates were identified with the Phoenix™ M50 system, and antimicrobial susceptibility testing was conducted by Phoenix™ M50 and disk diffusion, interpreted according to Comité de l’Antibiogramme de la Société Française de Microbiologie (CA-SFM)/European Committee on Antimicrobial Susceptibility Testing (EUCAST) 2025 guidelines. Categorical variables were expressed as counts and percentages; comparisons of proportions were made using the chi-square test or Fisher’s exact test when appropriate, with p < 0.05 considered significant.

Results

Among 469 specimens, 269 yielded bacterial growth (culture positivity rate 57.35%), corresponding to 309 isolates. Most culture positive patients were male (84.01%) and between 60-80 years (55.39%); 92.6% were inpatients. Fine needle aspirates had a higher positivity rate than swabs (60.1% vs 44.2%; p = 0.01). Infections were mainly monomicrobial (83.27%). Gram-negative organisms were the most frequent (66.66%), while gram-positive organisms represented 33.33%. The most frequent pathogens were *Escherichia coli* (24.59%), *Staphylococcus aureus* (19.74%), *Pseudomonas aeruginosa* (12.62%), and *Klebsiella pneumoniae* (9.70%). The prevalence of methicillin-resistant *Staphylococcus aureus* (MRSA) in *S. aureus* was 14.75%; resistance to penicillin G among staphylococci was very high, whereas all staphylococcal and enterococcal isolates remained susceptible to vancomycin and linezolid. Among Enterobacterales (n = 163), 33.74% were resistant to third-generation cephalosporins, and 20.85% expressed an extended-spectrum β-lactamase-producing (ESBL) phenotype; AmpC production was detected in 12.88% overall and in 43.47% of AmpC prone-species. Carbapenem resistance was observed in 24 Enterobacterales (14.72%). In *P. aeruginosa*, resistance to ceftazidime and/or carbapenems was modest (7.69%), and all isolates were susceptible to amikacin and colistin, whereas 75.0% of *Acinetobacter* spp. showed extensive drug resistance, with colistin as the only active agent.

Conclusions

The present study shows that pus-forming infections were dominated by Gram-negative organisms and showed a high burden of ESBL, AmpC and carbapenem-resistant Enterobacterales, contrasting with relatively preserved susceptibility in *P. aeruginosa* and extensive drug resistance in *Acinetobacter* spp. Fine-needle aspirates outperformed superficial swabs for pathogen detection. These findings support the use of deep sampling whenever feasible, regular updating of local antibiograms, and rational use of antimicrobial agents, particularly regarding third-generation cephalosporins and carbapenems, to optimize empirical therapy for pus-forming infections in this setting.

## Introduction

Pus-forming infections, such as abscesses and wound infections, are common in hospitals and other healthcare facilities and require prompt and appropriate antimicrobial therapy. These infections often prolong hospital stay, increase healthcare costs, and in severe cases, may progress to sepsis or chronic disability when not managed adequately. At the same time, the global spread of antimicrobial resistance (AMR) is limiting the efficacy of empirical therapy. The World Health Organization (WHO) now recognises AMR as a major public health threat, and recent global burden analyses indicate that resistant bacteria are already responsible for millions of deaths each year and may cause up to 10 million deaths annually by 2050 if no effective interventions are taken [1-5]. This threat is particularly concerning in low and middle-income countries, where inappropriate antibiotic use, restricted access to advanced agents, and insufficient laboratory infrastructure further complicate patient management [2-4].

Suppurative infections are associated with a wide variety of pathogens, from Gram-positive cocci to Gram-negative bacilli. *Staphylococcus aureus*, including methicillin-resistant *S. aureus* (MRSA), is a leading cause of skin and soft-tissue infections in both community and hospital settings, whereas gram-negative bacilli such as *Escherichia coli*, *Klebsiella pneumoniae* and *Pseudomonas aeruginosa* are increasingly involved, particularly in deep, polymicrobial or healthcare-associated infections [6-10]. In surgical wards, intensive care units, and diabetic foot clinics, these organisms are frequently isolated from pus, wound exudates, and drainage material, reflecting their ability to form biofilms and persist in damaged tissues or on medical devices [6-9]. The emergence of MRSA, extended-spectrum β-lactamase (ESBL) and AmpC-producing Enterobacterales, as well as carbapenem-resistant organisms, greatly limits therapeutic options and makes clinical management more challenging [2,7,11-15]. In such contexts, accurate microbiological documentation is essential to distinguish colonisation from true infection and to support the de-escalation of broad-spectrum antibiotic regimens.

In Morocco and across North Africa, published data on the microbiology and resistance patterns of pus-forming infections remain relatively limited and fragmented, despite the growing number of reports describing extended-spectrum beta-lactamase (ESBL)-producing Enterobacterales, MRSA, and other multidrug-resistant organisms in hospital settings [3,4,12-14]. Advanced care hospitals, particularly those with surgical and critical care units, represent key observatories for monitoring local AMR trends and optimising empirical antibiotic policies. The objective of this study was to characterize the bacterial profile and antimicrobial resistance patterns of isolates recovered from pus specimens (superficial wound swabs and fine-needle aspirates) in an advanced care hospital in Marrakech, Morocco, by describing the main pathogens and predominant resistance phenotypes. This work aims to generate locally relevant data to guide empirical therapy, support infection prevention strategies, and complement ongoing national and international AMR surveillance efforts.

## Materials and methods

Study design and setting

We conducted a single-centre, cross-sectional observational study in the microbiology laboratory of the Avicenna Military Hospital in Marrakech, Morocco. The objective was to describe the bacterial profile and antimicrobial resistance patterns of pus specimens, including superficial wound swabs and fine-needle aspirates. The study covered a 16-month period, from July 2024 to October 2025.

Study population, inclusion, and exclusion criteria

Clinical specimens were obtained from patients of both sexes and all age groups, regardless of whether they were hospitalised in medical or surgical wards or managed as outpatients.

Inclusion Criteria

We included all patients with clinically suspected deep or superficial skin and soft tissue infection for whom a pus specimen (fine-needle aspiration or wound swab) was sent to the microbiology laboratory during the study period.

Exclusion Criteria

Specimens were excluded if they were not properly collected or transported (leaking or unlabelled containers, major transport delay, missing or inconsistent patient identifiers), if they did not contain visible pus (dry swabs or purely serous exudates), or if they represented duplicate samples or isolates from the same patient and the same episode of infection. In such cases, only the first isolate was retained for analysis.

Specimen collection and transport

A total of 469 clinical specimens (fine needle aspiration and wound swabs) were received by the microbiology laboratory. The choice of specimen type was made by the treating clinician based on lesion depth and the presence of a collection. Fine-needle aspirates were preferentially obtained for deep-seated collections/abscesses or when surface sampling was likely to be contaminated by skin flora, whereas swabs were mainly used for superficial open wounds. This clinical sampling approach may influence the observed pathogen distribution. Specimens were collected under aseptic conditions using sterile devices and transported to the laboratory as quickly as possible after collection. When immediate processing could not be ensured, specimens were kept at 4 °C during transport and then cultured on arrival.

Microbiological procedures

Direct Microscopic Examination

For each specimen, a smear was prepared, stained by Gram, and examined under oil immersion (×100 objective lens). The microscopic examination was used to assess the presence and morphology of bacteria and inflammatory cells and to support the interpretation of subsequent culture and susceptibility results.

Culture and Identification of Isolates

Specimens were inoculated onto conventional culture media, including blood agar, chocolate agar, and mannitol salt agar (Chapman medium), and incubated at 37°C under aerobic conditions with 5% of CO_2_ for 24 hours. Deep specimens were also inoculated onto Schaedler agar and blood agar supplemented with colistin and nalidixic acid and incubated under anaerobic conditions at 37°C for 48 hours. Bacterial growth was evaluated based on colony morphology. A new Gram stain and basic orientation tests (oxidase and catalase) were performed on isolated colonies, and additional biochemical tests were added when required. Final identification to the genus and species level was obtained using the Phoenix™ M50 automated system (Becton Dickinson, Singapore).

Antimicrobial Susceptibility Testing

Testing methods: Antimicrobial susceptibility testing (AST) was primarily performed using the Phoenix™ M50 automated system. When required to confirm or further characterise resistance phenotypes, additional testing was carried out by the agar disk diffusion method. For disk diffusion, bacterial suspensions were adjusted to a turbidity equivalent to 0.5 McFarland and inoculated as a lawn onto agar plates. Antibiotic disks were then applied, and plates were incubated at 37 °C for 18-22 hours. Inhibition zone diameters and minimum inhibitory concentrations (MICs) generated by Phoenix™ 50 were interpreted according to the 2025 recommendations of Comité de l’Antibiogramme de la Société Française de Microbiologie (CA-SFM)/European Committee on Antimicrobial Susceptibility Testing (EUCAST) 2025. For Enterobacterales, carbapenem resistance was defined as non-susceptibility (I or R, according to CASFM/EUCAST 2025) to at least one carbapenem tested (ertapenem, imipenem, and/or meropenem).

Extended-spectrum β-lactamase (ESBL) production in Enterobacterales was initially screened by the Phoenix™ M50 system and systematically confirmed by a clavulanate synergy test, using ceftazidime and cefotaxime disks with and without clavulanic acid placed in proximity on Mueller-Hinton agar, in accordance with CASFM/EUCAST 2025 recommendations. Isolates showing a ≥5 mm increase in inhibition zone diameter in the presence of clavulanate were classified as ESBL producers and considered to exhibit an ESBL phenotype.

Among staphylococci, methicillin resistance was assessed using cefoxitin as a surrogate marker. Cefoxitin susceptibility was determined by the Phoenix™ M50 system and/or disk diffusion, and isolates of *Staphylococcus aureus *or coagulase-negative staphylococci (CoNS) categorised as resistant to cefoxitin were considered methicillin-resistant (MRSA and MR-CoNS) according to CASFM/EUCAST 2025 criteria.

Antimicrobial Agents Tested

**Figure 1:**
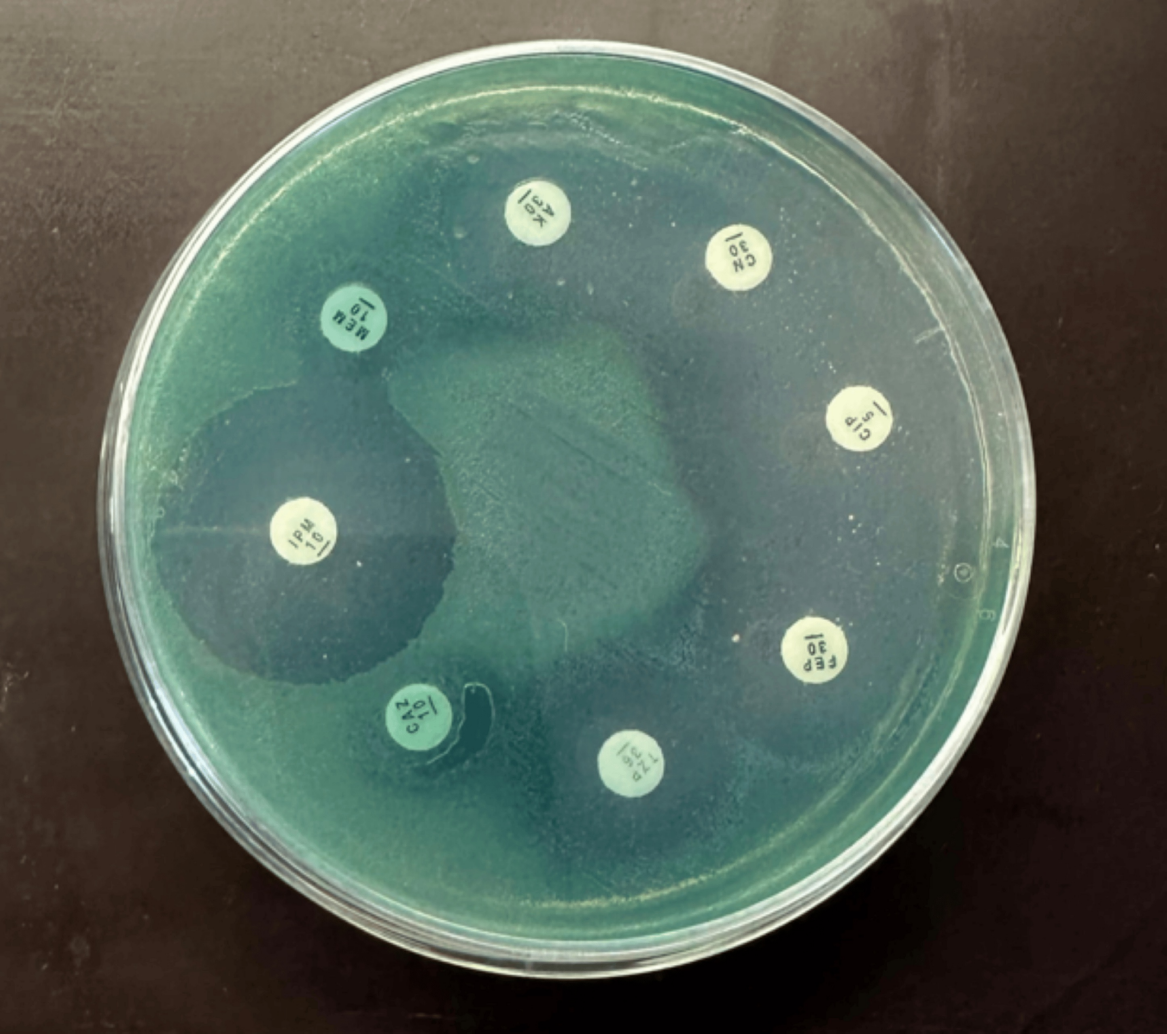
Antimicrobial susceptibility testing for non-fermenting Gram-negative bacilli Non-fermenting gram-negative bacilli: *Pseudomonas aeruginosa* on Mueller-Hinton agar; Sensitive: imipenem (IMP), cefepime (FEP), ciprofloxacin (CIP), amikacin (AK), gentamicin (CN); Resistant: ceftazidime (CAZ), piperacillin-tazobactam (TZP), meropenem (MEM).

For Gram-positive organisms, the antimicrobial panel included fusidic acid, amikacin, gentamicin, penicillin G, cefoxitin, ciprofloxacin, clindamycin, erythromycin, tetracycline, linezolid, vancomycin and co-trimoxazole (trimethoprim/sulfamethoxazole). For Gram-negative organisms, including Enterobacterales and non-fermenting Gram-negative bacilli (Figure 1), the following agents were tested as appropriate: gentamicin, amikacin, amoxicillin, amoxicillin-clavulanate, piperacillin-tazobactam, ceftazidime, ceftriaxone, cefepime, ertapenem, imipenem, meropenem, ciprofloxacin, tigecycline, co-trimoxazole (trimethoprim/sulfamethoxazole), and colistin. Colistin susceptibility was assessed using minimum inhibitory concentration (MIC) results provided by the Phoenix™ 50 system; disk diffusion was not used for colistin.

Quality control

Quality control was ensured using the following reference strains: Escherichia coli ATCC 25922, *Staphylococcus aureus* ATCC 25923, and *Pseudomonas aeruginosa* ATCC 27853, according to CASFM/EUCAST 2025 guidelines

Statistical analysis

Data were analysed using SPSS Statistics, version 31.0.1.0 (IBM Corp., Armonk, NY). Data tabulation and figures were prepared using Microsoft Excel (Microsoft Corporation, Redmond, WA). Categorical variables were expressed as counts and percentages. Comparisons of proportions were carried out using the Chi-square test or Fisher’s exact test when appropriate. A p-value of less than 0.05 was considered statistically significant. All analyses and figures were produced by the authors using standard office software (Microsoft Excel) and did not rely on proprietary scoring systems or copyrighted scales requiring specific permission or licensing.

## Results

Demographic and clinical characteristics

Table 1 summarizes the characteristics of the study population. During the study period, 469 purulent specimens (fine-needle aspirates and wound swabs) were collected, of which 269 yielded bacterial growth, corresponding to an overall culture positivity rate of 57.35%. Most culture-positive patients were aged 60-80 years (55.39%), followed by those aged 40-60 years (27.50%) and 20-40 years (17.10%). Male patients predominated, representing 84.01% (n = 226) of cases, whereas females accounted for 15.98% (n = 43). 

**Table 1:** Demographic characteristics of patients with culture positive pus specimens

Characteristic	Frequency (n)	Percentage (%)
Age group (years)		
20 to 40	46	17.10
40 to 60	74	27.50
60 to 80	149	55.39
Gender		
Male	226	84.01
Female	43	15.98

Table 2 shows that the majority of culture-positive specimens originated from inpatients, who accounted for 92.56% (n = 249) of cases, whereas only 7.43% (n = 20) were obtained from outpatients.

**Table 2:** Distribution of patients according to care setting

Patient setting	Number (n)	Percentage (%)
Inpatient	249	92.56
Outpatient	20	7.43

Sample type and infection pattern

**Figure 2:**
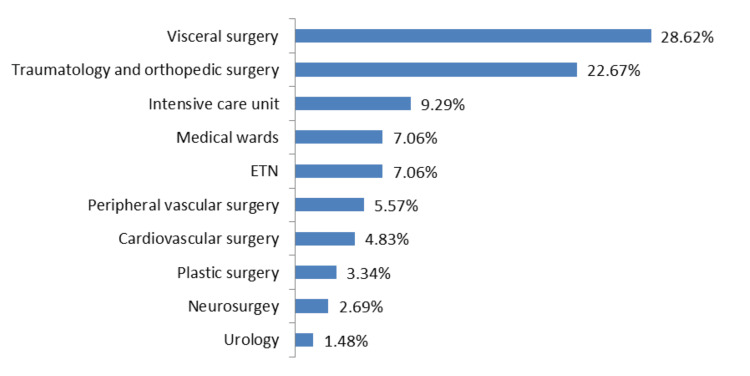
Distribution of clinical specimens by hospital department ENT: Ear, nose, throat.

The majority of specimens came from surgical wards. Visceral surgery was the main contributor (28.62%), followed by traumatology and orthopedic surgery (22.67%) and the intensive care unit (9.29%). Medical wards and ear, nose, throat (ENT) each accounted for (7.06%) of samples, peripheral vascular surgery for (5.57%), cardiovascular surgery for (4.83%), plastic surgery for (3.34%), neurosurgery for (2.69%), and urology for (1.48%) as shown in Figure 2.

Table 3 shows that fine-needle aspirates yielded a higher culture positivity rate (59.94%) than wound swabs (44.15%), and the difference was statistically significant (p = 0.01).

**Table 3:** Culture positivity by specimen type (swab vs fine-needle aspiration) n: Number; %: Percentage.

Specimen type	Positive specimens (n)	Total specimens (N)	Positivity rate (%)	Statistical test	Test statistic	p-value
Swab	34	77	44.15	Pearson Chi-square	χ² = 6.563 (df = 1)	0.01
Fine-needle aspiration	235	392	59.94			
Total	269	469	57.35			

Table 4 shows the distribution of monomicrobial and polymicrobial infections by patient setting. Of the 269 culture-positive cultures, 83.27%(n=224) represented monomicrobial infections and 16.72% (n=45) polymicrobial infections. The distribution of monomicrobial versus polymicrobial infections did not differ significantly between inpatients and outpatients (p = 0.754).

**Table 4:** Distribution of monomicrobial and polymicrobial infections by patient setting n: Number; %: Percentage; OR: Odds ratio.

Type of infection	Inpatients, n (%)	Outpatients, n (%)	Total, n (%)	Statistical test	Test statistic	p-value
Monomicrobial	208 (83.53%)	16 (80.00%)	224 (83.27%)	Fisher’s exact test	OR = 1.27 (Outpatients vs Inpatients)	0.754
Polymicrobial	41 (16.46%)	4 (20.00%)	45 (16.72%)			
Total	249 (92.56%)	20 (7.43%)	269 (100%)			

Bacterial spectrum

Table 5 summarizes the distribution of gram-negative and gram-positive isolates. Overall, 309 bacterial isolates were recovered from 269 culture-positive specimens. Gram-negative bacilli predominated, accounting for 66.66% (n = 206) of isolates, whereas gram-positive cocci represented 33.33% (n = 103).

**Table 5:** Distribution of organisms according to gram stain

Gram nature	Total isolate (n)	Percentage (%)
Gram-negative	206	66.66%
Gram-positive	103	33.33%
Total	309	100%

Among the 309 isolates, Enterobacterales accounted for 52.75% (n = 163), including *Escherichia coli *24.59% (n = 76), *Klebsiella pneumoniae* 9.70% (n = 30), *Enterobacter cloacae* 7.11% (n = 22), *Serratia marcescens* 2.91% (n = 9), *Proteus* spp. 2.58% (n = 8), *Morganella morganii *2.26% (n = 7), *Klebsiella aerogenes* 1.61% (n = 5), *Citrobacter* spp. 1.61% (n = 5) and *Providencia rettgeri* 0.32% (n = 1). Staphylococci represented 22.30% (n = 69) of isolates, mainly *Staphylococcus aureus *19.74% (n = 61) and coagulase-negative staphylococci (CoNS) 2.58% (n = 8). *Streptococcus* spp. and *Enterococcus* spp. accounted for 8.09% (n = 25) and 2.91% (n = 9), respectively, while *Pseudomonas aeruginosa* represented 12.62% (n = 39) and *Acinetobacter* spp. 1.29% (n = 4) of all isolates. These distributions are summarized in Table 6.

**Table 6:** Distribution of bacterial isolates by bacterial group and species GAS: Group A streptococcus; CBS: Group B streptococcus; GCS: Group C streptococcus; n: Number; %: Percentage.

Bacterial species	Isolates (n)	Within-group (%)	Overall, % (n = 309)
Enterobacterales (n = 163)			
Escherichia coli	76	46.62%	24.59%
Klebsiella pneumoniae	30	18.40%	9.70%
Enterobacter cloacae	22	13.49%	7.11%
Serratia marcescens	9	5.52%	2.91%
Proteus mirabilis	6	3.68%	1.94%
Proteus vulgaris	2	1.22%	0.64%
Morganella morganii	7	4.29%	2.26%
Klebsiella aerogenes	5	3.06%	1.61%
Citrobacter koseri	3	1.84%	0.97%
Citrobacter freundii	2	1.22%	0.64%
Providencia rettgeri	1	0.61%	0.32%
Staphylococci (n = 69)			
Staphylococcus aureus	61	88.40%	19.74%
Coagulase-negative staphylococci (CoNS)	8	11.59%	2.58%
Streptococci (n = 25)			
*Streptococcus pyogenes *(GAS)	11	44%	3.55%
*Streptococcus agalactiae *(GBS)	9	36%	2.91%
*Streptococcus dysgalactia *(GCS)	2	8%	0.64%
Streptoccocus anginosus	3	12%	0.97%
Enterococci (n = 9)			
Enterococcus faecium	6	66.66%	1.94%
Enterococcus faecalis	3	33.33%	0.97%
Pseudomonas (n = 39)			
Pseudomonas aeruginosa	39	100.00%	12.62%
Acinetobacter (n = 4)			
Acinetobacter baumannii	3	75%	0.97%
Acinetobacter lwoffi	1	25%	0.32%

As shown in Table 7, the bacterial distribution was similar between fine-needle aspiration and swab specimens. Enterobacterales remained the predominant group in both specimen types (52.69% in fine-needle aspirates vs. 53.06% in swabs; p = 0.962). The proportions of *Staphylococcus aureus* (19.23% vs. 22.45%; p = 0.604), coagulase-negative staphylococci (CoNS) (2.31% vs. 4.08%; p = 0.617), *Streptococcus* spp. (7.31% vs. 12.24%; p = 0.254), *Enterococcus* spp. (2.69% vs. 4.08%; p = 0.638), *Pseudomonas aeruginosa *(11.92% vs. 16.33%; p = 0.395) and *Acinetobacter *spp. (1.54% vs. 0%; p = 1.000) were also similar between the two sampling methods. Overall, none of the bacterial groups showed a statistically significant association with the sampling technique (all p-values > 0.05).

**Table 7:** Comparison of bacterial distribution between fine-needle aspiration and swab n: Number; %: Percentage; OR: Odds ratio. P-values were calculated using the Chi-square test (χ², df=1) or Fisher’s exact test (two-tailed) when expected cell counts were <5.

Bacterial group	Fine-needle aspiration (n=260), n (%)	Swab (n=49), n (%)	Statistical test	Test statistic	p-value
Enterobacterales	137 (52.69%)	26 (53.06%)	Pearson Chi-square	χ² = 0.002 (df = 1)	0.962
Staphylococcus aureus	50 (19.23%)	11 (22.45%)	Pearson Chi-square	χ² = 0.270 (df = 1)	0.604
Coagulase-negative staphylococci (CoNS)	6 (2.31%)	2 (4.08%)	Fisher’s exact test	OR = 0.56	0.617
Streptococcus spp.	19 (7.31%)	6 (12.24%)	Fisher’s exact test	OR = 0.57	0.254
Enterococcus spp.	7 (2.69%)	2 (4.08%)	Fisher’s exact test	OR = 0.65	0.638
Pseudomonas aeruginosa	31 (11.92%)	8 (16.33%)	Pearson Chi-square	χ² = 0.725 (df = 1)	0.395
Acinetobacter spp.	4 (1.54%)	0 (0.00%)	Fisher’s exact test	OR = non estimable	1.000

Antimicrobial resistance of gram-positive isolates

Table 8 presents antimicrobial resistance of S*taphylococcus aureus* and coagulase-negative staphylococci (CoNS). The proportion of MRSA was 14.75% among *S. aureus* isolates. Compared with *S.aureus*, coagulase-negative Staphylococci (CoNS) exhibited higher resistance rates to most antibiotics. Resistance to fusidic acid was 87.5% in CoNS versus 19.67% in *S. aureus* (p<0.001), methicillin-resistance reached 75.0% in CoNS versus 14.75% in *S. aureus* (p<0.001), and tetracycline resistance was 62.5% in CoNS versus 19.67% in *S. aureus* (p = 0.013); all three differences were statistically significant. CoNS also tended to show higher resistance to erythromycin (50.0% vs 22.95%), ciprofloxacin (37.5% vs 13.11%), amikacin (25.0% vs 11.47%), gentamicin (25.0% vs 4.91%), and co-trimoxazole (25.0% vs 8.19%). In both *S. aureus* and CoNS, almost all isolates were resistant to penicillin G (96.72% and 100%, respectively). By contrast, all Staphylococcal isolates remained fully susceptible to linezolid and vancomycin.

**Table 8:** Antimicrobial resistance profiles of Staphylococcus aureus and coagulase negative Staphylococci (CoNS) isolated from pus specimens %: Percentage; CoNS: Coagulase-negative Staphylococci. P-values were calculated using Fisher’s exact test (two-tailed) comparing resistance rates between *Staphylococcus aureus* and CoNS.

Antibiotic	* Staphylococcus aureus *(n = 61)		CoNS (n = 8)		p-value
	Sensitive	Resistant	Sensitive	Resistant	
Fusidic acid	50 (81.96%)	11 (19.67%)	1 (12.5%)	7 (87.5%)	<0.001
Amikacin	54 (88.52%)	7 (11.47%)	6 (75%)	2 (25%)	0.278
Gentamicin	58 (95.08%)	3 (4.91%)	6 (75%)	2 (25%)	0.099
Penicillin G	2 (3.27%)	59 (96.72%)	0 (0%)	8 (100%)	1.000
Cefoxitin	52 (85.24%)	9 (14.75%)	2 (25%)	6 (75%)	<0.001
Ciprofloxacin	53 (86.88%)	8 (13.11%)	5 (62.5%)	3 (37.5%)	0.109
Clindamycin	52 (85.24%)	9 (14.75%)	7 (87.5%)	1 (12.5%)	1.000
Erythromycin	47 (77.04%)	14 (22.95%)	4 (50%)	4 (50%)	0.192
Linezolid	61 (100%)	0 (0%)	8 (100%)	0 (0%)	1.000
Vancomycin	61 (100%)	0 (0%)	8 (100%)	0 (0%)	1.000
Tetracycline	50 (81.96%)	11 (19.67%)	3 (37.5%)	5 (62.5%)	0.013
Co-trimoxazole	56 (91.80%)	5 (8.19%)	6 (75%)	2 (25%)	0.184

*Streptococcus* spp. overall retained high susceptibility to the tested agents, except *Streptococcus agalactiae*, which showed a high level of resistance to tetracycline (71.42%). Among Enterococci, *Enterococcus faecium* exhibited a less favorable susceptibility profile than *Enterococcus faecalis*. Resistance to gentamicin, tetracycline and ciprofloxacin was approximately twice as high in *E. faecium* (66.66% each) as in* E. faecalis* (33.33% each). All *E. faecium* isolates were resistant to ampicillin (100%), whereas *E. faecalis* remained fully susceptible. However, both species were uniformly susceptible to vancomycin and linezolid.

Antimicrobial resistance of gram-negative isolates

Enterobacterales

Table 9 shows the antimicrobial resistance pattern of Enterobacterales. *E. coli* showed high resistance to amoxicillin-clavulanate 61.84%, co-trimoxazole 44.74% and ciprofloxacin (36.84%). In *K. pneumoniae*, resistance was particularly high to amoxicillin-clavulanate 73.33%, ceftriaxone 70.0%, cefepime and piperacillin-tazobactam 60.0% each, with carbapenem resistance exceeding 40%. *E. cloacae* exhibited elevated resistance to ceftriaxone and co-trimoxazole, 45.45% each, as well as to piperacillin-tazobactam 40.91% and ciprofloxacin 36.36%. In *Proteus* spp., resistance was highest to amoxicillin 75.0%, whereas resistance to most other agents remained low. *M. morganii *showed substantial resistance to tigecycline 71.43%, and 42.86% of isolates were resistant to gentamicin, piperacillin-tazobactam, ceftriaxone, ciprofloxacin, and co-trimoxazole. *K. aerogenes* showed very high resistance to piperacillin-tazobactam and ceftriaxone (80.0% each), while *Citrobacter* spp. were notably resistant to amoxicillin-clavulanate (80.0%) and co-trimoxazole (40.0%). Across all Enterobacterales, amikacin was the most active agent, with 93.25% of isolates remaining susceptible.

**Table 9:** Antimicrobial resistance profiles of Enterobacterales isolated from pus specimens *E. coli: Escherichia coli; K. pneumoniae: Klebsiella pneumoniae; E. cloacae: Enterobacter cloacae; S. marcescens: Serratia marcescens; Proteus *spp*: Proteus *species*; M. morganii: Morganella morganii; K. aerogenes: Klebsiella aerogenes; Citrobacter *spp:​​​ *Citrobacter *species *indicates an antimicrobial to which the species is considered intrinsically resistant.

Antibiotic	*E. coli* (n= 76)	*K. pneumoniae *(n=30)	*E. cloacae* (n=22)	*S. marcescens* (n=9)	*Proteus* spp (n=8)	*M. morganii *(n=7)	*K. aerogenes *(n=5)	*Citrobacter* spp (n=5)
Gentamicin	8 (10.53%)	10 (33.33%)	4 (18.18%)	0 (0%)	0 (0%)	3 (42.86%)	0 (0%)	1 (20%)
Amikacin	4 (5.26%)	2 (6.67%)	2 (9.09%)	1 (11.11%)	0 (0%)	1 (14.29%)	1 (20%)	0 (0%)
Amoxicillin	61 (80.26%)	30 (100%)*	22 (100%)*	9 (100%)*	6 (75%)	7 (100%)*	5 (100%)*	5 (100%)*
Amoxicillin-clavulanate	45 (61.84%)	22 (73.33%)	22 (100%)*	9 (100%)*	2 (25%)	7 (100%)*	5 (100%)*	4 (80%)
Piperacillin-tazobactam	25 (32.89%)	18 (60%)	9 (40.91%)	2 (22.22%)	1 (12.5%)	3 (42.86%)	4 (80%)	0 (0%)
Ceftriaxone	17 (22.37%)	21 (70%)	10 (45.45%)	2 (22.22%)	1 (12.5%)	3 (42.86%)	4 (80%)	1 (20%)
Cefepime	14 (18.42%)	18 (60%)	4 (18.18%)	1 (11.11%)	1 (12.5%)	0 (0%)	1 (20%)	0 (0%)
Ertapenem	2 (2.63%)	13 (43.33%)	5 (22.73%)	1 (11.11%)	0 (0%)	0 (0%)	2 (40%)	0 (0%)
Meropenem	1 (1.32%)	12 (40%)	3 (13.64%)	1 (11.11%)	0 (0%)	0 (0%)	1 (20%)	0 (0%)
Ciprofloxacin	28 (36.84%)	19 (63.33%)	8 (36.36%)	0 (0%)	1 (12.5%)	3 (42.86%)	2 (40%)	0 (0%)
Tigecycline	5 (6.58%)	13 (43.33%)	7 (31.82%)	0 (0%)	1 (12.5%)	5 (71.43%)	1 (20%)	0 (0%)
Co-trimoxazole	34 (44.74%)	12 (40%)	10 (45.45%)	1 (11.11%)	1 (12.5%)	3 (42.86%)	1 (20%)	2 (40%)

Non-fermenting Gram-negative Bacilli

Table 10 summarizes resistance patterns of *Pseudomonas aeruginosa* (n = 39) and *Acinetobacter* spp. (n = 4). Among *P. aeruginosa* isolates, resistance rates were 7.69% for gentamicin, 12.82% for piperacillin-tazobactam, 10.25% for cefepime, 7.69% for ceftazidime, 5.12% for imipenem, 5.12% for meropenem, and 17.94% for ciprofloxacin. No resistance to amikacin or colistin was observed. Among *Acinetobacter* spp. resistance was detected in 75% of isolates to gentamicin, amikacin, piperacillin-tazobactam, cefepime, ceftazidime, imipenem, meropenem, and ciprofloxacin. For tigecycline and co-trimoxazole, resistance reached 50% each, while no colistin resistance was recorded.

**Table 10:** Antimicrobial resistance profiles of non-fermenting gram-negative bacilli isolated from pus specimens *Acinetobacter* spp: *Acinetobacter* species *indicates antimicrobial to which the species is intrinsically resistant

Antibiotic	* Pseudomonas aeruginosa* (n = 39)		*Acinetobacter *spp (n = 4)	
	Sensitive	Resistant	Sensitive	Resistant
Gentamicin	36 (92.30%)	3 (7.69%)	1 (25%)	3 (75%)
Amikacin	39 (100%)	0 (0%)	1 (25%)	3 (75%)
Piperacillin-tazobactam	34 (87.17%)	5 (12.82%)	1 (25%)	3 (75%)
Cefepime	35 (89.74%)	4 (10.25%)	1 (25%)	3 (75%)
Ceftazidime	36 (92.30%)	3 (7.69%)	1 (25%)	3 (75%)
Imipenem	37 (94.87%)	2 (5.12%)	1 (25%)	3 (75%)
Meropenem	37 (94.87%)	2 (5.12%)	1 (25%)	3 (75%)
Ciprofloxacin	32 (82.05%)	7 (17.94%)	1 (25%)	3 (75%)
Tigecycline	0 (0%)	39 (100%)*	2 (50%)	2 (50%)
Co-trimoxazole	0 (0%)	39 (100%)*	2 (50%)	2 (50%)
Colistin	39 (100%)	0 (0%)	4 (100%)	0 (0%)

Prevalence of extended-spectrum β-lactamase (ESBL), AmpC-producing and carbapenem resistance among Enterobacterales

Table 11 shows the distribution of ESBL and carbapenem resistance among Enterobacterales. Of the 163 Enterobacterales isolated from purulent specimens, 55 isolates (33.74%) were resistant to third-generation cephalosporins. Phenotypic ESBL production was confirmed in 34 isolates, corresponding to 20.85% of all Enterobacterales and to 61.81% (34/55) of third-generation cephalosporin-resistant strains. At the species level, ESBL production was observed in 16/76 *Escherichia coli* (21.05%), 17/30 *Klebsiella pneumoniae* (56.66%), and 1/8 *Proteus* spp. (12.5%).In addition to ESBL producers, 21 isolates (12.88%) were classified as AmpC-producing Enterobacterales. Among AmpC-prone species (*Enterobacter cloacae, Serratia marcescens, Morganella morganii, Klebsiella aerogenes, Citrobacter freundii, Providencia rettgeri*, n = 46), AmpC production accounted for 43.47% (20/46). Carbapenem resistance (defined as non-susceptibility to ≥1 tested carbapenem) was detected in 24 Enterobacterales isolates, representing 14.72% of all Enterobacterales recovered. *Klebsiella pneumoniae *was the predominant species, accounting for more than half of all carbapenem-resistant isolates (13/24, 54.17%). The *Enterobacter cloacae* constituted the second most represented group (5/24, 20.83%), followed by *Escherichia coli* (2/24, 8.33%) and *Klebsiella aerogenes* (2/24, 8.33%). Isolated cases of carbapenem resistance were also documented in *Serratia marcescens* (1/24, 4.17%) and *Proteus* spp. (1/24, 4.17%).

**Table 11:** Distribution of ESBL production and carbapenem resistance among Enterobacterales isolates ESBL: Extended-spectrum beta-lactamase; n: Number; %: Percentage.

Species	ESBL productiong n (%)	Carbapenem resistant n (%)
Escherichia coli	16 (21.05%)	2 (2.63%)
Klebsiella pneumoniae	17 (56.66%)	13 (43.33%)
*Proteus* spp.	1 (12.5%)	1 (12.5%)
Enterobacter cloacae	0 (0%)	5 (22.72%)
Serratia marcescens	0 (0%)	1 (11.11%)
Klebsiella aerogenes	0 (0%)	2 (40.00%)
Total	34	24

Distribution of multidrug-resistant (MDR), extensively drug-resistant (XDR), and pan-drug-resistant (PDR) phenotypes in Gram-positive and Gram-negative isolates

Among gram-positive bacteria, *Staphylococcus aureus* accounted for two MDR isolates out of 61 (3.27%), with no XDR strains. Coagulase-negative staphylococci (CoNS) included 7 MDR isolates out of 8 (87.5%), and E*nterococcus faecium* showed 4 MDR isolates out of six (66.66%). No XDR phenotype was observed among gram-positive species and no PDR isolates were detected. Among gram-negative bacteria, Enterobacterales comprised 163 isolates, of which 68 (41.71%) were MDR, and 25 (15.33%) were XDR. *Pseudomonas aeruginosa* included three MDR isolates out of 39 (7.69%), with no XDR strains. *Acinetobacter* spp. comprised four isolates, three of which were XDR (75.0%). No pan-drug-resistant (PDR) isolates were detected among gram-negative bacteria. These distributions are shown in Table 12.

**Table 12:** Distribution of MDR, XDR, and PDR phenotypes among bacterial isolates Multi-drug-resistant (MDR), extensively drug-resistant (XDR), and pan-drug-resistant (PDR) were defined according to international consensus criteria [16], based on antimicrobial categories tested for each isolate. n: Number; %: Pecentage.

Bacterial group	MDR n (%)	XDR n (%)	PDR n (%)
Staphylococcus aureus	2 (3.27%)	0 (0%)	0 (0%)
Coagulase negative Staphylococci (CoNS)	7 (87.5%)	0 (0%)	0 (0%)
Enterococcus faecium	4 (66.66%)	0 (0%)	0 (0%)
Enterobacterales	68 (41.71%)	25 (15.33%)	0 (0%)
Pseudomonas aeruginosa	3 (7.69%)	0 (0%)	0 (0%)
*Acinetobacter* spp.	0 (0%)	3 (75.0%)	0 (0%)

## Discussion

In this study, we describe the microbiological profile of pus-forming infections in an advanced care hospital in Marrakech, Morocco, focusing on both the spectrum of pathogens and key resistance phenotypes. The study population was predominantly elderly and male: 55.39% of culture positive patients were between 60 and 80 years and 84.01% were men. These findings are in line with a diabetic foot cohorts, where the mean patient age was 59 years [8], but contrasts with an Algerian wound infection cohort, where the mean age was around 37.7 years and women were slightly more represented [6]. These differences probably reflect variations in referral patterns and case mix, with our hospital managing a higher proportion of older surgical patients with multiple comorbidities.

Our overall culture positivity rate was 57.35%, which was lower than the 65.65% reported in a North-Central Algerian wound-swab series and also lower than the rates described in Moroccan burn units and diabetic foot cohorts around 68% [6,8]. An important practical finding is the significantly higher yield obtained with fine-needle aspirates compared with superficial swabs (59.94% versus 44.15%; p = 0.01). This result contrasts with a previous study on diabetic foot infections, in which superficial swab cultures showed a higher positivity rate than deep samples (54.39% vs 45.60%) [8].In our series, the more frequent use of fine-needle aspiration (FNA) for deep purulent collections led to a higher proportion of positive FNA samples, which more accurately reflect true invasive infection. These findings support existing recommendations that deep, properly collected specimens should be preferred whenever feasible, in order to optimise pathogen recovery and minimise misinterpretation related to colonising flora [6,8].

The dominance of Gram-negative bacilli (66.66%) over Gram-positive cocci (33.33%) in our series differs from many classical descriptions of superficial skin and soft tissue infections, where *Staphylococcus aureus* frequently predominates [6,10,11]. Our distribution is more consistent with deep seated or healthcare associated infections and is comparable to Moroccan diabetic foot data, where gram-negative bacilli contribute substantially to the microbiological burden [8]. This interpretation is further supported by the predominance of deep specimens in the present study, as fine-needle aspirates are more likely to recover Gram-negative bacilli and resistant phenotypes than superficial swabs in surgical populations, and are less affected by surface colonization. In our cohort, *Escherichia coli *was the most common pathogen (24.59%), followed by *S. aureus* (19.74%), *Pseudomonas aeruginosa* (12.62%), and *Klebsiella pneumoniae* (9.70%). Similar pathogen rankings, particularly for *E. coli*, *K. pneumoniae,* and *P. aeruginosa*, have been reported in studies from Algeria, India, and Nepal [6,10,11]. In our series, *Staphylococcus aureus* was the second most frequently isolated pathogen, in keeping with previous studies reporting *S. aureus *as a major cause of suppurative infections [7,8]. The relatively low proportion of coagulase-negative staphylococci is probably related to careful interpretation and exclusion of likely contaminants.

Among gram-positive cocci, very high resistance to penicillin G was observed in both *S. aureus* (96.72%) and coagulase-negative staphylococci (100%), in line with widespread β-lactamase production described in regional and international series [6-8]. The proportion of methicillin-resistant *S. aureus* (MRSA) in our study was 14.75%. This level is clearly lower than that reported in a Nepalese study (around 60.6%) [11], and in Moroccan burn units (approximately 22%) [7], but exceeds the 4.7% MRSA rate documented in community-acquired diabetic foot infections in Rabat [8]. Despite these variations, a reassuring finding is that all staphylococcal and enterococcal isolates remained susceptible to vancomycin and linezolid, consistent with Moroccan reports indicating that glycopeptide resistance is still rare [1,8,9]. *Streptococcus* spp. remained highly susceptible to most antibiotics, except for *Streptococcus agalactiae*, which showed notable resistance to tetracycline (71.42%). This rate, however, was lower than the 94% resistance reported by Lahlou et al. [9].

The most worrisome aspect of our results concerns the resistance profile of gram-negative bacilli. Among Enterobacterales (n = 163), 55 isolates (33.74%) were resistant to third-generation cephalosporins. Within this subset, 34 isolates expressed an extended-spectrum β-lactamase (ESBL) phenotype, corresponding to 20.85% of all Enterobacterales and 61.81% of third-generation cephalosporin-resistant strains. These values are higher than the ESBL rate of 14.1% reported in Moroccan diabetic foot infections [8] and are compatible with the growing burden of ESBL-producing Enterobacterales described across North Africa [6]. This trend is also supported by Moroccan AMR synthesis data and surveillance reports highlighting ESBL as a major resistance phenotype among Enterobacterales [1]. In addition, studies from Moroccan hospitals, including reports from Marrakech, have documented a high rate of ESBL and the circulation of highly resistant Enterobacterales, further supporting the relevance of our findings in the local context [12-14]. AmpC production was also prominent in our series, particularly among AmpC-prone species, in which 43.47% of isolates were classified as AmpC producers, illustrating the complexity of resistance mechanisms that may not be fully identified by ESBL screening alone.

Carbapenem resistance among Enterobacterales reached 14.72% (23 isolates), exceeding the 9% prevalence documented by Belouad et al. in a Rabat hospital [14]. At the city level, work from Mohammed VI University Hospital in Marrakech has already reported the circulation of carbapenemase-producing *K. pneumoniae* and *Enterobacter cloacae*, mainly driven by OXA-48-like and NDM enzymes [12,13]. Systematic reviews from Africa further indicate that carbapenem-resistant Enterobacterales are increasingly implicated in severe infections and hospital outbreaks [15,17]. Taken together with global burden estimates, these findings align with the WHO’s designation of carbapenem-resistant enterobacterales as critical priority pathogens for antimicrobial development [18].

The situation among non-fermenting gram-negative bacilli was more contrasted. In *P. aeruginosa*, resistance remained relatively limited: only 7.69% of isolates were resistant to ceftazidime and/or carbapenems, and all were susceptible to amikacin and colistin. These data are similar to Moroccan diabetic foot results and suggest that, at least in our setting, *P. aeruginosa* has not yet reached the very high resistance levels reported in some other tertiary care centres [7,8,10]. Conversely, *Acinetobacter *spp. displayed a particularly unfavourable profile, with 75.0% of isolates classified as extensively drug-resistant, with only colistin retaining complete activity. This pattern is compatible with extensively drug-resistant *A. baumannii* phenotypes described in Moroccan burn units and in other North African hospitals [3,7,8].

Overall, our findings highlight a setting in which the MRSA rate is intermediate compared with the wide variability reported across the region (very high rates in burn units and in some tertiary care wound series, but lower rates in other Moroccan cohorts) [7-9,11]. Glycopeptide activity also appears preserved against gram-positive cocci in Moroccan surveillance and hospital reports, consistent with the absence of vancomycin or linezolid resistance observed in our isolates [1,8,9]. In addition, *Pseudomonas aeruginosa* showed acceptable susceptibility in our series, in line with Moroccan diabetic foot data and comparable pus/wound studies from other settings [8,10]. By contrast, Enterobacterales in our hospital exhibited a high burden of ESBL, AmpC, and carbapenem resistance, which is in line with Moroccan AMR syntheses, reports documenting carbapenemase-producing Enterobacterales in Marrakech and other Moroccan hospitals, as well as with broader African data on carbapenem-resistant Enterobacterales [1,12,17]. Finally, *Acinetobacter* spp. were frequently extensively drug-resistant, a pattern that aligns with Moroccan ICU and burn unit reports describing difficult-to-treat *A. baumannii *infections [3,7]. Taken together, these findings underscore the need to strengthen infection prevention and control and antimicrobial stewardship, prioritizing the rational use of third-generation cephalosporins and carbapenems in line with the broader public health imperative to limit the spread of AMR and focus on critical priority-resistant pathogens [2,5,18]. 

Limitations

Selection bias related to specimen type is possible, as fine-needle aspirates constituted most samples and may over-represent deep-seated and/or healthcare-associated infections; therefore, generalizability to cohorts dominated by superficial wound swabs may be limited. In addition, confirmatory carbapenemase testing was not systematically available, limiting characterization of carbapenem non-susceptibility mechanisms.

## Conclusions

In this advanced care hospital in Morocco, pus-forming infections were mainly caused by gram-negative bacilli, particularly *Escherichia coli*, *Klebsiella pneumoniae* and *Pseudomonas aeruginosa*, with *Staphylococcus aureus* as the leading gram-positive pathogen. Fine-needle aspiration performed better than superficial swabbing for pathogen recovery, which supports the systematic use of deep sampling whenever possible to improve microbiological diagnosis. Among gram-positive organisms, *S. aureus* showed an intermediate prevalence of methicillin-resistance, and both staphylococci and enterococci remained fully susceptible to vancomycin and linezolid. The most concerning result is the substantial burden of multidrug-resistant Enterobacterales, including ESBL and AmpC-producing isolates and a notable proportion of carbapenem resistance. Susceptibility remained relatively preserved in *P. aeruginosa*, whereas *Acinetobacter* spp. frequently displayed extensively-drug-resistant profiles, leaving colistin as the only reliable therapeutic option.

Taken together, these results show that there is an urgent need to strengthen infection prevention and control, to keep local antibiograms up to date, and to apply targeted antimicrobial stewardship, especially for third-generation cephalosporins and carbapenems. It is essential to choose better empirical treatments for pus-forming infections and to slow the spread of resistance in similar hospitals.
